# 50565 CTSA collaboration to support K-12 school re-opening in the COVID-19 pandemic

**DOI:** 10.1017/cts.2021.610

**Published:** 2021-03-31

**Authors:** Moira Inkelas, Onyebuchi A. Arah, Vladimir G. Manuel, Roch Nianogo, Douglas E. Morrison, Nathaniel Anderson, Defne Yilmaz, Tony Kuo

**Affiliations:** 1 Fielding School of Public Health, University of California Los Angeles; 2 David Geffen School of Medicine, University of California Los Angeles

## Abstract

ABSTRACT IMPACT: The mobilization of a CTSA-sponsored team with multi-disciplinary translational science expertise enabled the university to provide a range of T1-T4 expertise to a large, complex school district that resulted in permanent learning and data science infrastructure. OBJECTIVES/GOALS: The Clinical Translational Science Institute (CTSI) formed a multidisciplinary science team to provide expertise in support of the re-opening of in-person learning in the second-largest U.S. school district during the COVID-19 pandemic. METHODS/STUDY POPULATION: The assembled interdisciplinary science team provided expertise in epidemiology, machine learning, causal inference and agent-based modeling, data and improvement science, biostatistics, clinical and laboratory medicine, health education, community engagement, and experience in outbreak investigation and management. The team included TL1 pre and postdoctoral fellows and mobilized scientists from multiple professional schools and T1-T4 stages of translational research. RESULTS/ANTICIPATED RESULTS: Tangible outcomes achieved using this team approach included the development of practical metrics for use in the school community, a learning process, the integration of preventive design elements into a testing and tracing program, and targeted and data-driven health education. The team, for example, generated new data displays for community engagement and collaborated with the school district in their use to visualize, learn from, and act on variation across a 700 square mile region. DISCUSSION/SIGNIFICANCE OF FINDINGS: Novel translational methods can be used to establish a learning environment and data science infrastructure that complements efforts of public health agencies to aid schools in the COVID-19 pandemic. These new capabilities apply to COVID-19 testing and vaccines and can be mobilized for future population health challenges faced by school districts.

